# Antiproliferation and Induction of Apoptosis in Ca9-22 Oral Cancer Cells by Ethanolic Extract of *Gracilaria tenuistipitata*

**DOI:** 10.3390/molecules170910916

**Published:** 2012-09-11

**Authors:** Chi-Chen Yeh, Chao-Neng Tseng, Jing-Iong Yang, Hurng-Wern Huang, Yi Fang, Jen-Yang Tang, Fang-Rong Chang, Hsueh-Wei Chang

**Affiliations:** 1 Graduate Institute of Natural Products, Kaohsiung Medical University, Kaohsiung 807, Taiwan; 2 Department of Biomedical Science and Environmental Biology, Kaohsiung Medical University, Kaohsiung 807, Taiwan; 3 Department of Seafood Science, National Kaohsiung Marine University, Kaohsiung 811, Taiwan; 4 Institute of Biomedical Science, National Sun Yat-Sen University, Kaohsiung 804, Taiwan; 5 Department of Radiation Oncology, Faculty of Medicine, College of Medicine, Kaohsiung Medical University, Kaohsiung 807, Taiwan; 6 Department of Radiation Oncology, Kaohsiung Medical University Hospital, Kaohsiung 807, Taiwan; 7 Cancer Center, Kaohsiung Medical University Hospital, Kaohsiung Medical University, Kaohsiung 807, Taiwan

**Keywords:** oral cancer, apoptosis, ROS, glutathione, mitochondrial membrane potential, marine natural product

## Abstract

The water extract of *Gracilaria tenuistipitata* have been found to be protective against oxidative stress-induced cellular DNA damage, but the biological function of the ethanolic extracts of *G. tenuistipitata* (EEGT) is still unknown. In this study, the effect of EEGT on oral squamous cell cancer (OSCC) Ca9-22 cell line was examined in terms of the cell proliferation and oxidative stress responses. The cell viability of EEGT-treated OSCC cells was significantly reduced in a dose-response manner (*p* < 0.0001). The annexin V intensity and pan-caspase activity of EEGT-treated OSCC cells were significantly increased in a dose-response manner (*p* < 0.05 to 0.0001). EEGT significantly increased the reactive oxygen species (ROS) level (*p* < 0.0001) and decreased the glutathione (GSH) level (*p* < 0.01) in a dose-response manner. The mitochondrial membrane potential (MMP) of EEGT-treated OSCC cells was significantly decreased in a dose-response manner (*p* < 0.005). In conclusion, we have demonstrated that EEGT induced the growth inhibition and apoptosis of OSCC cells, which was accompanied by ROS increase, GSH depletion, caspase activation, and mitochondrial depolarization. Therefore, EEGT may have potent antitumor effect against oral cancer cells.

## 1. Introduction

Oral squamous cell cancer (OSCC) has high morbidity and mortality rates across the world because it is frequently found in advanced stages before therapy [1,2]. The conventional strategies of OSCC management still depend on surgery, radiotherapy, chemotherapy and targeted therapy [3]. The poor outcome of chemotherapy to OSCC contributes to the poor prognosis for OSCC [4]. Therefore, novel, effective therapy for OSCC treatment is still needed.

Marine natural products provide abundant resources for antitumor drug discovery [5,6]. Recently, algae preparations have become a popular treatment in alternative medicine. *Gracilaria* algae have been cultivated in Taiwan for at least 50 years [7] and are abundant and cheap and used in natural medicines. Many species of *Gracilaria* algae are well established to be a potential source for drug discovery in natural medicines due to their antibacterial, antiviral, antifungal, antihypertensive, cytotoxic, spermicidal, embriotoxic, and anti-inflammatory effects [8]. However, the species *Gracilaria tenuistipitata* in Taiwan is not included in this review. Therefore, we were interested in the biological effects of different extracts of *G. tenuistipitata*. Previously, we have demonstrated that the water extracts of *G. tenuistipitata* can reduce the hydrogen peroxide-induced oxidative DNA damage [9]. However, the cellular response to the ethanol extracts of *G. tenuistipitata* (EEGT) was still unknown. Hence, in this study the biological effects for ethanolic extracts of EEGT on oral cancer cells were examined. We evaluated the possible antiproliferative effects against OSCC (Ca9-22) cells by EEGT as well as its possible mechanism involving apoptosis and oxidative stress.

## 2. Results

### 2.1. Cytotoxicity Effects of EEGT-Treated Ca9-22 Oral Cancer Cells

**Figure 1:**
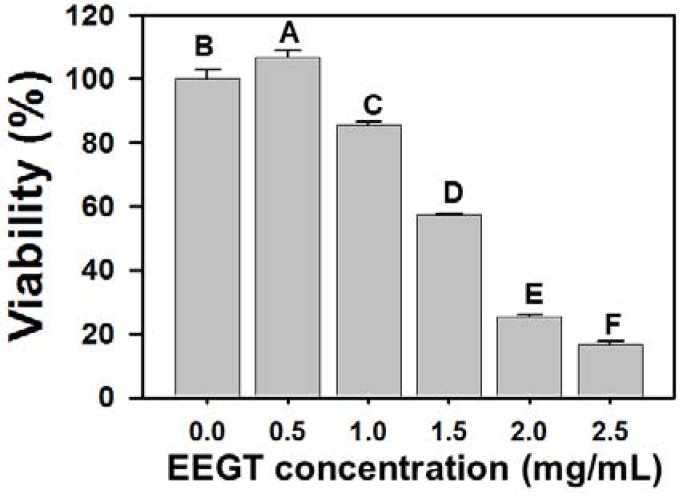
Proliferation of Ca9-22 oral cancer cells is inhibited by ethanolic extracts of *G. tenuistipitata* (EEGT). Cells were incubated with various concentrations of EEGT (0, 0.5, 1, 1.5, 2 and 2.5 mg/mL) for 24 h. Cell viability was determined by MTS assay. Data are expressed as mean ± S.D. (n = 6). Differences between treatments of different concentrations containing the same capital letter at the top of each column are not significant.

In the MTS assay (Figure 1), the relative cell viability at various concentrations of EEGT (0, 0.5, 1, 1.5, 2 and 2.5 mg/mL) after 24 h were 100.0 ± 2.8, 106.7 ± 2.2, 85.5 ± 1.2, 57.5 ± 0.4, 25.3 ± 0.7 and 16.8 ± 1.1 (n = 6). The cell viability of EEGT-treated Ca9-22 oral cells significantly decreased in a dose-response manner (*p* < 0.0001).

### 2.2. Apoptosis Induction of EEGT-Treated Ca9-22 Oral Cells

**Figure 2:**
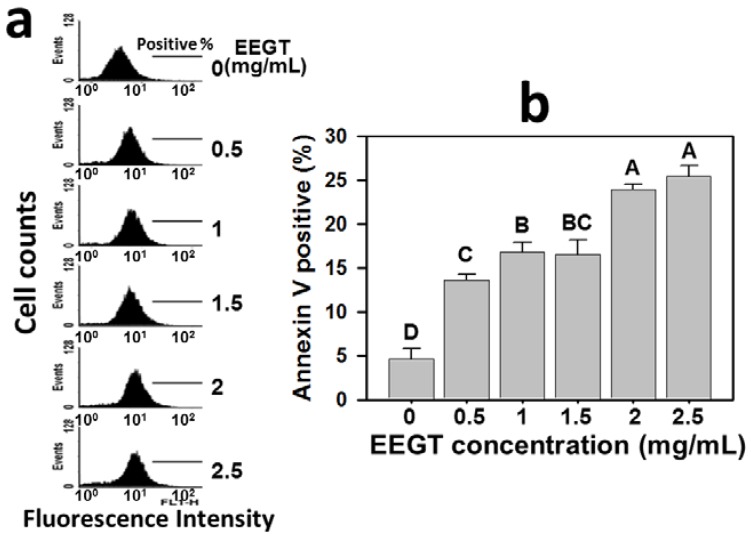
Ethanolic extracts of *G. tenuistipitata* (EEGT) induced apoptosis of Ca9-22 oral cancer cells. (**a**) Cells treated with different concentrations (0 to 2.5 mg/mL) of EEGT for 24 h were stained with annexin V-FITC. Positive % is indicated in each panel; (**b**) Quantificative analysis of annexin V-positive population. Data are presented as mean ± S.D. (n = 3). Differences between treatments of different concentrations containing the same capital letter at the top of each column are not significant.

In Figure 2a, the profiles of annexin V-positive percentages were shown for the treatments with vehicle control or 0.5, 1, 1.5, 2 and 2.5 mg/mL of EEGT for 24 h. After 24 h EEGT treatment, the annexin V-positive percentages of Ca9-22 oral cancer cells were significantly increased in a dose-response manner for most concentrations (*p* < 0.05 to 0.0001) (Figure 2b).

### 2.3. Activation of Pan-Caspase in EEGT-Treated Ca9-22 Oral Cancer Cells

**Figure 3:**
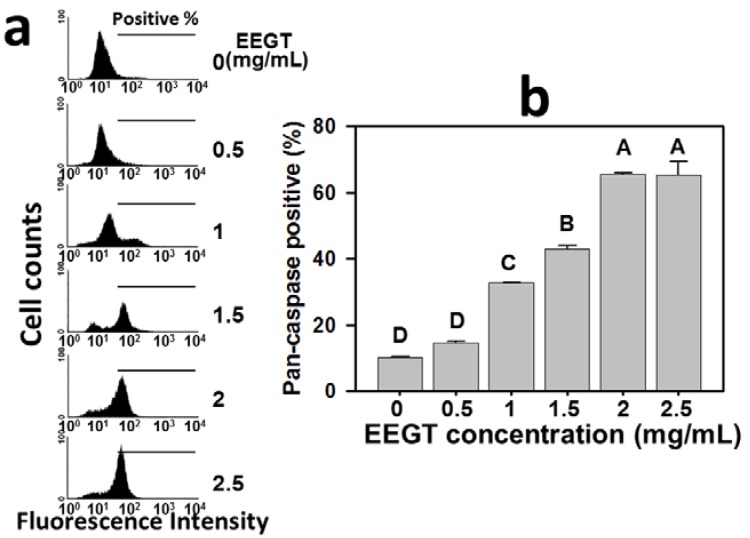
Ethanolic extracts of *G. tenuistipitata* (EEGT) induced activation of generic caspase in Ca9-22 oral cancer cells. (**a**) Cells treated with different concentrations (0 to 2.5 mg/mL) of EEGT for 24 h were stained with 1 μL 500X TF2-VAD-FMK. Positive % is indicated in each panel; (**b**) Quantificative analysis of pan-caspase fluorescent intensity. Data are presented as mean ± S.D. (n = 3). Differences between treatments of different concentrations containing the same capital letter at the top of each column are not significant.

The role of caspases in the EEGT-induced apoptosis of Ca9-22 oral cancer cells was examined by the flow cytometry-based TF2-VAD-FMK assay (Figure 3). The pan-caspase activities were increased at concentrations from 0 to 2.5 mg/mL EEGT (Figure 3a). Apparently, the generic caspase activities in cells treated with EEGT ranging from 0.5 to 2 mg/mL showed a significant increase in a dose-response manner (*p* < 0.0001) (Figure 3b).

### 2.4. Induction of Reactive Oxygen Species (ROS) in EEGT-Treated Ca9-22 Oral Cancer Cells

**Figure 4:**
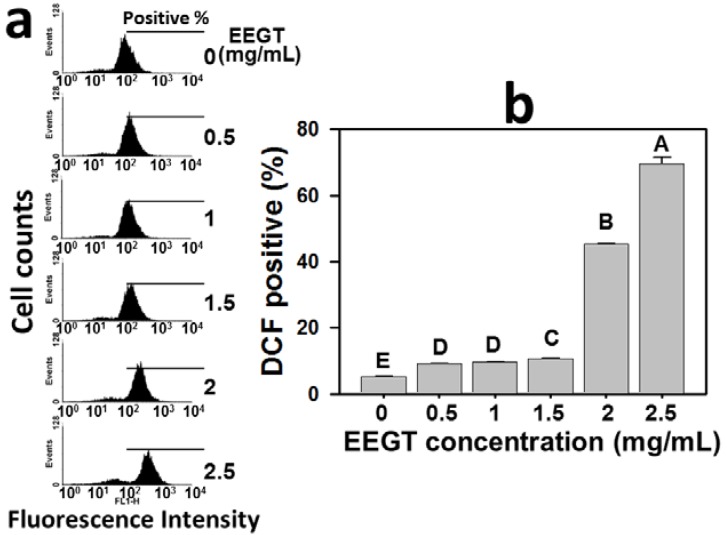
Ethanolic extracts of *G. tenuistipitata* (EEGT) increased reactive oxygen species (ROS) levels of Ca9-22 oral cancer cells. (**a**) Flow cytometry-based ROS profiles for EEGT-treated cells. Cells treated with different concentrations (0 to 2.5 mg/mL) of EEGT for 24 h. Positive % is indicated in each panel; (**b**) Quantificative analysis of DCF-positive population. Data are presented as mean ± S.D. (n = 3). Differences between treatments of different concentrations containing the same capital letter at the top of each column are not significant.

The role of ROS in the EEGT-induced apoptosis of Ca9-22 oral cancer cells was examined by the flow cytometry-based DCFH-DA assay (Figure 4). The profiles of ROS-positive percentages of 0, 0.5, 1, 1.5, 2 and 2.5 mg/mL EEGT for 24 h were shown (Figure 4a). The ROS-positive percentages of EEGT-treated Ca9-22 oral cancer cells were significantly increased in a dose-response manner after 1.5 mg/mL (*p* < 0.0001) (Figure 4b).

### 2.5. Depletion of Intracellular Reduced Glutathione (GSH) in EEGT-Treated Ca9-22 Oral Cancer Cells

**Figure 5:**
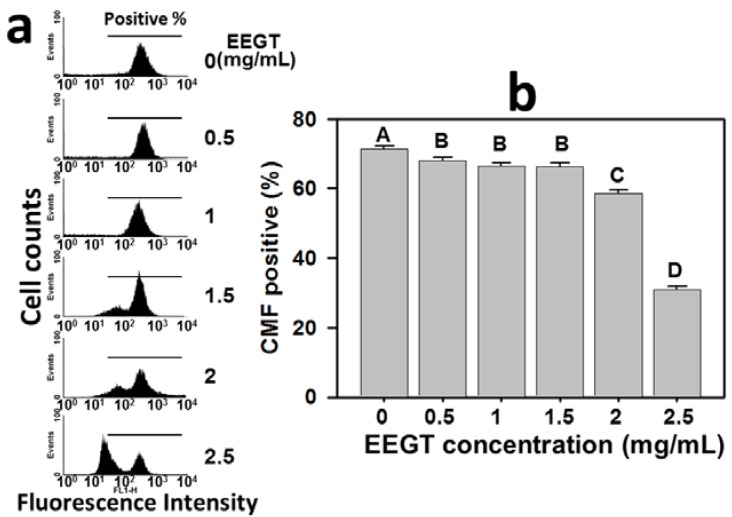
Ethanolic extracts of *G. tenuistipitata* (EEGT) induced glutathione (GSH) depletion of Ca9-22 oral cancer cells. (**a**) Flow cytometry-based GSH profiles for EEGT-treated cells. Cells treated with different concentrations (0 to 2.5 mg/mL) of EEGT for 24 h. Positive % is indicated in each panel; (**b**) Quantificative analysis of CMF-positive %. Data are presented as mean ± S.D. (n = 3). Differences between treatments of different concentrations containing the same capital letter at the top of each column are not significant.

The role of GSH in the EEGT-induced ROS change of Ca9-22 oral cancer cells was examined by the flow cytometry-based CMF-DA assay. The profiles of GSH-positive percentages of 0, 0.5, 1, 1.5, 2 and 2.5 mg/mL EEGT for 24 h were shown (Figure 5a). The GSH-positive percentages of EEGT-treated Ca9-22 oral cancer cells were significantly reduced in a dose-response manner (*p* < 0.01) (Figure 5b).

### 2.6. Mitochondrial Membrane Potential (MMP) Decrease in EEGT-Treated Ca9-22 Oral Cancer Cells

**Figure 6:**
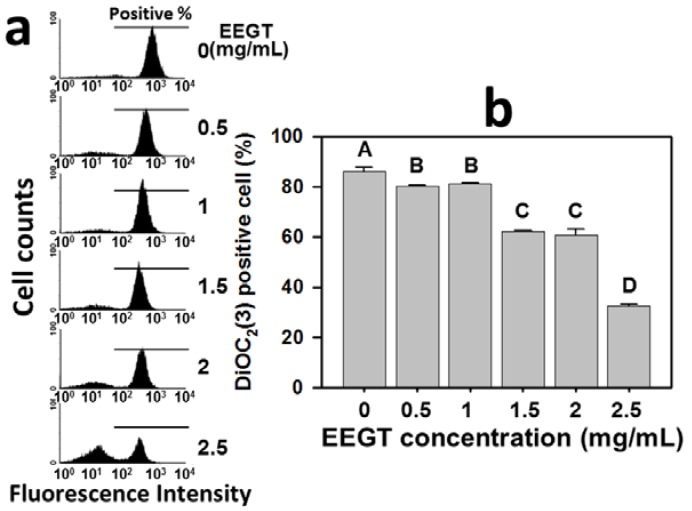
Ethanolic extracts of *G. tenuistipitata* (EEGT) reduced mitochondrial membrane potential (MMP) in Ca9-22 oral cancer cells. (**a**) Flow cytometry-based MMP profiles for EEGT-treated cells. Cells treated with different concentrations (0 to 2.5 mg/mL) of EEGT for 24 h. Positive % is indicated by horizontal lines; (**b**) Quantificative analysis of DiOC_2_(3) intensity. Data are presented as mean ± S.D. (n = 3). Differences between treatments of different concentrations containing the same capital letter at the top of each column are not significant.

The role of MMP in the EEGT-induced ROS change of Ca9-22 oral cancer cells was examined by the flow cytometry-based DiOC_2_(3) assay. The profiles of DiOC_2_(3)-positive percentages of 0, 0.5, 1, 1.5, 2 and 2.5 mg/mL EEGT for 24 h were shown (Figure 6a). The DiOC_2_(3)-positive percentages of EEGT-treated Ca9-22 oral cancer cells was significantly reduced in a dose-response manner (*p* < 0.005) (Figure 6b).

### 2.7. Discussion

In our previous work, we found that the water extracts of *G. tenuistipitata* (AEGT) reduced hydrogen peroxide-induced oxidative DNA damage [9]. In this study, we further demonstrated that EEGT has antiproliferative effects against Ca9-22 oral cancer cells in a dose-dependent manner. To reduce possible toxic effect to normal cells under the current dosage, future work with the active fraction may reduce the dosage of the EEGT. We have found that AEGT has more vitamin C content than EEGT (data not shown) and it may contribute to the higher anti-oxidative activity of AEGT. Similar to other natural products, the water extract of *Portulaca oleracea* was reported to significantly inhibit DNA damage, while its ethanolic extract had no effect [10]. These results suggest that different extraction reagents may result in different formulations that generate different functions [11].

Similar to our finding of the anti-oral cancer effect of EEGT, several ethanolic extracts of natural products have demonstrated potential antiproliferative effects in cancer; such as *Corydalis yanhusuo* against breast cancer [12], *Dunaliella salina*, *Spirulina platensis*, and *Aphanizomenon flos-aquae* against leukemic cells [13], propolis against cervical cancer [14], *Corchorus olitorius* against liver cancer [15], and *Scutellaria baicalensis* against lung cancer [16]. However, some ethanolic extracts of natural products may display chemopreventive effects towards cancer rather than cytotoxic effects as described above. For example, the ethanolic extract of the red algae *Laurencia tristicha* possesses antioxidative activity and decreases DNA damage [17]. The ethanolic extract of the brown algae *Sargassum dentifolium* has potential hepatoprotective effects [18]. Therefore, the antiproliferative effects of ethanolic extracts of natural products seem to vary between species.

Modulating the cell death associated pathways is a successful strategy for cancer therapy [19,20,21,22]. For example, many natural product-derived compounds have demonstrated an apoptosis-related antiproliferative effect on cancer, such as protoapigenone [23] and 4β-hydroxywithanolide E [24] for lung cancer cells, dryofragin [25] for breast cancer cells, and berberine [26], and goniothalamin [27] for oral cancer cells. Similarly, we found that the annexin V staining intensity of Ca9-22 oral cancer cells was increased in a dose-dependent manner (Figure 2). Both extrinsic and intrinsic pathways of apoptosis have been reported to converge at the level of the effector caspases [28,29]. To further confirm the involvement of apoptosis, the caspase activation in EEGT-treated oral cancer cells was performed. The general caspase activities were increased in a dose-dependent manner for EEGT treatment (Figure 3). Therefore, the EEGT-induced antiproliferative effect in OSCC cells may be partly due to apoptosis.

Cancer cells have an increased basal oxidative stress [30]. The high levels of ROS in cancer cells make cancer cells sensitive to treatments that further increase ROS levels [31]. The strategy of modulating the oxidative stress to drug discovery for anti-cancer studies has been proposed [31,32]. Accordingly, the present study validated this strategy by the finding that EEGT induced the ROS generation in OSCC cells in a dose-response manner (Figure 4). Moreover, some anticancer therapies have demonstrated that GSH depletion can increase ROS generation and further induces apoptosis in glioma [33], leukemia [34], and colon cancer [35]. In the same fashion, we found that GSH was depleted in the EEGT-treated oral cancer cells in a dose-response manner (Figure 5). These results suggest that the oxidative stress plays an important role in the antiproliferative effect of EEGT against OSCC cells.

It has been shown that ROS induction can depolarize the mitochondrial membrane potential, which eventually increases the expression of pro-apoptotic molecules [36,37]. For example, ergocalciferol induced apoptosis of leukemia cells by causing mitochondria dysfunction through ROS production, GSH depletion, caspase activation, and Fas induction [38]. Similarly, the present study demonstrated that EEGT significantly decreased the MMP in OSCC cells in a dose-response manner, suggesting that EEGT-induced mitochondria dysfunction may modulate the oxidative stress and lead to apoptosis in OSCC cells.

## 3. Experimental

### 3.1. Raw Materials and Ethanol Extract Preparation

The fresh seaweed *Gracilaria tenuistipitata* was collected in spring of 2009 from a marine culture farm at Kouhu Beach, Yunlin County, Taiwan. The dried sample was prepared as described previously [9]. Dried samples (50 g) were soaked in ethanol (250 mL) three times and extracted with 1,000 mL of 99.9% ethanol with shaking at room temperature for 24 h. Then the ethanol extract solution was filtered with Whatman No. 1 filter paper three times and evaporated to dryness at 40 ± 2 °C on a rotary evaporator (Buchi Laboratoriums-Technik, Flawil, Switzerland). The dry extract was stored in a sealed container at −20 °C until use.

### 3.2. Cell Cultures

The human OSCC cancer cell line Ca9-22 [27] was cultured in DMEM medium (Gibco, Grand Island, NY, USA) and supplemented with 10% fetal bovine serum (FBS), 100 U/mL penicillin, 100 μg/mL streptomycin, 0.03% glutamine and 1 mM sodium pyruvate. The cells were maintained at 37 °C in a humidified atmosphere containing 5% CO2.

### 3.3. Cell Viability Assay

EEGT was dissolved in DMSO and added to the medium. The final concentration of DMSO was less than 1%. The effects of EEGT on cell viability was estimated by the 3-(4,5-dimethylthiazol-2-yl)-(3-carboxymethoxyphenyl)-2-(4-sulphophenyl)-2H-tetrazolium (MTS) assay (CellTiter 96 AQueous One Solution, Promega, Madison, WI, USA) [39]. Briefly, cells were plated at a density of 1 × 10^5^ cells/well in a 96-well cell culture plate and treated with EEGT at doses of 0.5, 1, 1.5, 2 and 2.5 mg/mL for 24 h. After incubation, the MTS solution was added to cells (10 μL per well) and continued to incubate for 1–2 h at 37 °C. The absorbance at 490 nm was measured using Dynex MRX Model 96 Well Plate Reader (MTX Lab Systems, Inc., Vienna, VA, USA).

### 3.4. Apoptosis Assay

Apoptosis was measured by annexin V kit (Pharmingen, San Diego, CA, USA) as previously described [40]. Briefly, cells were treated with vehicle or increasing concentrations of EEGT for 24 h. Then, cells were incubated with 10 μg/mL of annexin V-fluorescein isothiocyanate (FITC) and analyzed using the FACSCalibur flow cytometer.

### 3.5. Pan-Caspase Activity Assay

The generic activation of caspases (Caspase-1, 3, 4, 5, 6, 7, 8, 9) was measured by the generic caspase activity assay kit (Abcam, Cambridge, UK). Most caspases have substrate selectivity for the peptide sequence Val-Ala-Asp (VAD). Nontoxic TF2-VAD-FMK is a fluorescent reporter for most caspase activities because it is cell permeable and irreversibly binds to these activated caspases. Briefly, EEGT-treated Ca9-22 cells were suspended in 0.5 mL warm medium at a density of approximately 1 × 10^6^ cells/mL, added 1 μL of 500X TF2-VAD-FMK, and incubated at 37 °C, 5% CO_2_, for 1 h. Cells were washed with PBS twice and resuspended in 0.5 mL of assay buffer for immediate measurement using flow cytometry with excitation and emission settings of 480 and 525 nm, respectively.

### 3.6. Intracellular Reactive Oxygen Species (ROS) Assay

Intracellular ROS levels were measured using 2',7'-dichlorodihydrofluorescein diacetate (DCFH-DA) as previously described [41]. Briefly, EEGT-treated cells were washed with PBS twice and then mixed with 10 μM H2DCF-DA in PBS for 30 min at 37 °C in the CO_2_ incubator. Cells were collected and washed twice with PBS. After centrifugation, cells were resuspended in PBS and immediately measured by the FACSCalibur flow cytometer with excitation and emission settings of 480 and 525 nm, respectively.

### 3.7. Intracellular Reduced Glutathione (GSH) Assay

Intracellular GSH was measured using 5-chloromethylfluorescein diacetate (CMF-DA) as previously described [41]. Briefly, EEGT-treated cells were incubated with 5 μM CMF-DA for 20 min at 37 °C in the CO_2_ incubator. After washing with PBS, cells were harvested by centrifugation, and then measured with the FACS-Calibur flow cytometer.

### 3.8. Mitochondrial Membrane Potential Assay

Mitochondrial membrane potential (MMP) was examined using a MitoProbe™ DiOC_2_(3) assay kit (Invitrogen, San Diego, CA, USA). Briefly, EEGT-treated Ca9-22 cells were suspended in 1 mL of warm PBS at approximately 1 × 10^6^ cells/mL, loaded with 5 μL of 10 μM DiOC_2_(3), and incubated at 37 °C in the CO_2_ incubator for 20 to 30 min. Subsequently, cells were collected, washed and resuspended in PBS for immediate analysis using a flow cytometry assay with excitation and emission settings of 480 and 525 nm, respectively.

### 3.9. Statistical Analysis

All data are presented as mean ± S.D. Comparison between experimental groups was assessed by one-way ANOVA with Tukey’s HSD Post Hoc Test using the software JMP^®^ 9 software. Differences between treatments of different concentrations containing the same capital letter are not significant.

## 4. Conclusions

In this study, we have demonstrated that EEGT has an antiproliferative effect against oral cancer cells by induction of apoptosis and the modulation of oxidative stress. These findings not only suggest that EEGT is a promising natural product extract for potential use in oral cancer therapy, but also demonstrate that finding compounds capable of inducing apoptosis and ROS is a promising strategy for anti-oral cancer drug discovery.
